# The impact of routine HIV drug resistance testing in Ontario: A controlled interrupted time series study

**DOI:** 10.1371/journal.pone.0246766

**Published:** 2021-04-02

**Authors:** Lawrence Mbuagbaw, Carmen H. Logie, Lehana Thabane, Fiona Smaill, Marek Smieja, Ann N. Burchell, Beth Rachlis, Jean-Eric Tarride, Abigail Kroch, Tony Mazzulli, Elizabeth Alvarez, Daeria O. Lawson, Francis Nguyen, Richard Perez, Hsien Seow

**Affiliations:** 1 Department of Health Research Methods, Evidence and Impact, McMaster University, Hamilton, ON, Canada; 2 Biostatistics Unit, Father Sean O’Sullivan Research Centre, St Joseph’s Healthcare, Hamilton, ON, Canada; 3 Centre for Development of Best Practices in Health (CDBPH), Yaoundé Central Hospital, Yaoundé, Cameroon; 4 Women’s College Research, Toronto, ON, Canada; 5 Factor-Inwentash Faculty of Social Work, University of Toronto, Toronto, ON, Canada; 6 Departments of Paediatrics and Anaesthesia, McMaster University, Hamilton, ON, Canada; 7 Centre for Evaluation of Medicine, St Joseph’s Healthcare, Hamilton, ON, Canada; 8 Population Health Research Institute, Hamilton Health Sciences, Hamilton, ON, Canada; 9 Department of Family and Community Medicine, Faculty of Medicine, University of Toronto, Toronto, ON, Canada; 10 Department of Family and Community Medicine and Centre for Research on Inner City Health, Li Ka Shing Knowledge Institute, St. Michael’s Hospital, Unity Health Toronto, Toronto, Ontario, Canada; 11 Ontario HIV Treatment Network, Toronto, Ontario, Canada; 12 Division of Clinical Public Health, Dalla Lana School of Public Health, University of Toronto, Toronto, Ontario, Canada; 13 McMaster Chair in Health Technology Management, McMaster University, Hamilton, ON, Canada; 14 Department of Laboratory Medicine and Pathobiology, Faculty of Medicine, University of Toronto, Toronto, Ontario, Canada; 15 Department of Microbiology, Sinai Health System/University Health Network, Toronto, Ontario, Canada; 16 Public Health Ontario Laboratory, Ontario, Canada; 17 Centre for Health Economics and Policy Analysis (CHEPA), McMaster University, Hamilton, Ontario, Canada; 18 ICES McMaster, McMaster University, Hamilton, Ontario, Canada; 19 Department of Oncology, McMaster University, Juravinski Cancer Centre, Hamilton, ON, Canada; Centers for Disease Control and Prevention, UNITED STATES

## Abstract

**Background:**

Knowledge of HIV drug resistance informs the choice of regimens and ensures that the most efficacious options are selected. In January 2014, a policy change to routine resistance testing was implemented in Ontario, Canada. The objective of this study was to investigate the policy change impact of routine resistance testing in people with HIV in Ontario, Canada since January 2014.

**Methods:**

We used data on people with HIV living in Ontario from administrative databases of the Institute for Clinical Evaluative Sciences (ICES) and Public Health Ontario (PHO), and ran ordinary least squares (OLS) models of interrupted time series to measure the levels and trends of 2-year mortality, 2-year hospitalizations and 2-year emergency department visits before (2005–2013) and after the policy change (2014–2017). Outcomes were collected in biannual periods, generating 18 periods before the intervention and 8 periods after. We included a control series of people who did not receive a resistance test within 3 months of HIV diagnosis.

**Results:**

Data included 12,996 people with HIV, of which 8881 (68.3%) were diagnosed between 2005 and 2013, and 4115 (31.7%) were diagnosed between 2014 and 2017. Policy change to routine resistance testing within 3 months of HIV diagnosis led to a decreasing trend in 2-year mortality of 0.8% every six months compared to the control group. No significant differences in hospitalizations or emergency department visits were noted.

**Interpretation:**

The policy of routine resistance testing within three months of diagnosis is beneficial at the population level.

## Introduction

There are approximately 36.9 million people living with HIV worldwide [1]. Antiretroviral therapy (ART) is the mainstay of HIV management and is recommended for everyone with HIV and for people at high risk of HIV infection as a prevention strategy [2]. The increasing use of ART has contributed to an increase in resistant strains of HIV [3]. Drug resistance severely hampers the effectiveness of ART, reduces the number of effective therapeutic options, increases infectiousness, and reduces quality of life and survival [4, 5]. Some people with drug resistant HIV are infected with a resistant virus, i.e. transmitted drug resistance (TDR), and this is a recognised global health problem [6]. People with TDR are at a higher risk of treatment failure, treatment discontinuation and developing new drug resistant strains [7]. The rise in drug resistance is one of the greatest threats to global health, and can result in millions of deaths, an increase in new harder-to-treat strains of HIV and higher health-care costs [8]. The prevalence of TDR varies worldwide, but is highest in North America at ~13% [9], likely due to the long-standing and widespread availability of ART. The prevalence of TDR in Canada (based on administrative data) has increased over time, from 9.5% to 27.3% (2002 to 2009) in Ontario [10] and 12% to 18% (1996 to 2016) in British Columbia [11].

Effectively dealing with TDR requires monitoring and timely response to population and individual levels of resistance. In high-income countries that can afford it, resistance testing is performed before the patient starts ART [12]. This approach allows physicians to determine the most efficacious combination of drugs for each patient [12]. However, the merits of this approach are uncertain. Some American studies suggest that drug resistance testing prior to initiating therapy has limited benefits and may not be cost-effective [13, 14].

Close to 71,000 people are living with HIV in Canada, of which 42% live in Ontario [15, 16], Canada’s most populous province. Ontario also accounts for the largest number of new cases of HIV [17]. In Ontario, resistance testing prior to initiating ART became available for physicians around 2005 (discretionary resistance testing), but a formal recommendation for resistance testing to be used as standard of care was only made in January 2014 (routine resistance testing) by Public Health Ontario (PHO) [18]. This policy change led to a corresponding laboratory practice change, and since then, all newly diagnosed people with HIV in Ontario are expected to receive a genotypic resistance test (GRT) prior to initiating ART if their viral load is sufficiently high (>1000 copies/mL).

The aim of this study was to investigate the population-level impact of the resistance testing policy in Ontario by comparing outcomes in the cohort of people with HIV who started treatment between 2005 and 2013 (discretionary resistance testing) to outcomes in the cohort of people who started treatment in 2014 to 2017 (routine resistance testing).

## Methods

### Ethics

The use of data in this project was authorized under section 45 of Ontario’s Personal Health Information Protection Act, which does not require review by a Research Ethics Board. This project was conducted under section 45 of Ontario’s Personal Health Information Privacy Act and approved by the Privacy and Legal Office of ICES (formerly known as Institute for Clinical Evaluative Sciences. ICES is an independent, non-profit research institute whose legal status under Ontario’s health information privacy law allows it to collect and analyze health care and demographic data, without consent, for health system evaluation and improvement.

ICES is the health information custodian for this study and PHO houses the results of all HIV drug resistance tests in Ontario. All datasets were linked using unique encoded identifiers. All projects that make use of ICES data are assessed for re-identification risks prior to release, which ensures that research outputs do not contain information that could identify an individual. ICES policies and procedures are approved by the Information and Privacy Commissioner of Ontario, allowing ICES to receive personal health information of Ontarians without their consent for the purpose of evaluating, planning, or monitoring Ontario’s health system, or for the purpose of research. ICES’ last completed review was in 2017. ICES is currently undergoing a 2020 review by the Information and Privacy Commissioner (IPC) of Ontario.

Data were pulled on May 14, 2020.

### Design

We conducted a controlled interrupted time-series (ITS) analysis of a cohort of people with HIV created from linked administrative databases (from the ICES and laboratory information from PHO).

### Outcomes

We selected three patient important and policy relevant outcomes at two years: mortality, hospitalization rate, and emergency department visits among incident HIV cases per biannual period, measured as the fraction of incident HIV cases in each time interval who had the outcome of interest in the follow-up period (i.e., the proportion of incident HIV cases in each biannual period who had the outcome of interest).

### Cohort

Cohort entry criteria was all incident cases of HIV in Ontario. HIV infection was determined based on a validated algorithm built upon physician claims for HIV-related care [19]. The index event date was defined as the first contact with the health system for HIV care determined by a positive HIV test or lab request for viral load or resistance testing. Participants were followed up until outcomes occurred or up to December 2019. The cohort was divided into two groups defined by whether a resistance test was used to inform care. The first group had a resistance test done within three months of HIV diagnosis, the second group had a resistance test done beyond 3 months of HIV diagnosis or had no record of a resistance test. A viral load test within 3 months of HIV diagnosis is an indicator for linkage to care [20], which has been used in other Canadian studies [21, 22]. People with a viral load test beyond 3 months of diagnosis are assumed to not be optimally engaged in care.

### Statistical analysis

Sample size and power considerations are outlined in S1 Appendix. We used descriptive statistics to characterise the cohort of treatment-naïve people in the years prior to and following the implementation of the policy. Continuous data is described using means (standard deviation) or medians (quartile 1; quartile 3). Categorical data is described using counts (percentage). Age, sex, residence, and the Ontario Marginalization Index (ON-Marg) economic dependency quintiles are reported [23]. Given that these are population level data (and not a random sample), statistical testing may not be meaningful, as such p-values are not reported [24].

We compared outcomes (bi-annually) in people with HIV who initiated treatment before the policy (2005–2013) to outcomes in people who initiated treatment after the policy (2014–2017) adjusting for whether they received a resistance test within 3 months of HIV diagnosis or not, using a controlled ITS approach. The ITS is considered the “next” best strategy for interventions when randomization is not possible and clinical trial data do not exist. It is particularly useful for natural experiments and the evaluation of health policies [25]. However, other events occurring around the time of the intervention may influence outcomes. One way of addressing this concern is to include a control series. No effect in the control series provides stronger evidence in favor of the intervention [26]. In this context, it is expected that resistance tests conducted within 3 months of HIV diagnosis are used to inform first-line treatments and therefore the group of people who received a resistance test much later, or no resistance test at all, would provide an appropriate counterfactual, i.e. what would have happened if there was no policy change. In this analysis a control group is even more important given that before the policy, some people might have initiated treatment informed by a resistance test, and likewise after the policy, some people may not have received a resistance test prior to initiating treatment. Other Canadian studies have shown that access to resistance testing is not uniform [11, 27, 28]. However, ITS only informs inferences at the population level and we can not make inferences about individuals.

We used Ordinary Least Squares (OLS) models to assess the impact of the intervention on our outcomes of interest, both immediately and over time. The models included terms to evaluate the following variables: a constant to represent level of outcome (e.g. mortality) at baseline (before the policy), terms for the linear trend before the policy, the change in level of the outcome immediately after the policy, the change in trend after the policy, the difference in baseline level of outcome between treatment and control groups, the difference in trends between treatment and control before the policy, the difference in change in outcome between the treatment and control group immediately after the policy and the difference between treatment and control groups in the trend of the outcome variable after initiation of the intervention compared with before the policy.

For each modeled outcome of interest, we reported the maximum likelihood regression coefficient estimates from the final models, confidence intervals and p-values. The full ITS model and details on how we addressed potential autocorrelation is described in S2 Appendix and S3 Appendix, respectively.

These analyses were conducted with due consideration of sufficient equally spaced data points before and after the intervention, linearity of pre-intervention trends, stable measurement procedures and incorporate available data on a potential confounder (timely resistance testing) [29]. Trends are illustrated graphically. As a sensitivity analysis, we first ran similar models excluding outliers and secondly estimated our outcomes quarterly. Our findings are reported according to the Strengthening the Reporting of Observational Studies in Epidemiology (STROBE) guidelines [30]. All analyses were performed in SAS statistical software (version 9.4) and figures produced in Stata (version 16.0).

## Results

In this controlled ITS we included 18 points (bi-annual) before the intervention and 8 points after the intervention.

### Cohort characteristics

We included data from a total of 12,996 people with HIV, of which 8,881 (68.3%) were diagnosed between 2005 and 2013, and 4,115 (31.7%) were diagnosed between 2014 and 2017. In the pre-policy period 25.4% (2262/8881) received a resistance test early (within 3 months of diagnosis), compared to 28.7% (1183/4115) in the post-policy period. The distribution of age, sex, residence, and economic dependency are reported in Table 1.

**Table 1:** Characteristics of participants.

Variable	Pre-policy period (2005–2013)			Policy period			Total
				(2014–2019)			
	Late or no resistance testing**	Early resistance testing*	Total	Late or no resistance testing**	Early resistance testing *	Total	Grand Total
			(N = 8,881)			(N = 4,115)	
							(N = 12,996)
		(N = 2,262)			(N = 1,183)		
				(N = 2,932)			
	(N = 6,619)						
**Age: mean (SD)**	34.60 (18.57)	37.56 (11.25)	35.36 (17.05)	35.14 (18.30)	37.49 (12.42)	35.82 (16.85)	35.50 (16.99)
**Sex: n (%)**							
**Female**	2,071 (31.3)	338 (14.9)	2,409 (27.1)	790 (26.9)	161 (13.6)	951 (23.1)	3,360 (25.9)
**Male**	4,548 (68.7)	1,924 (85.1)	6,472 (72.9)	2,142 (73.1)	1,022 (86.4)	3,164 (76.9)	9,636 (74.1)
**Residence: n (%)**							
**Urban**	6,344 (95.8)	2,187 (96.7)	8,531 (96.1)	2,822 (96.2)	1,150 (97.2)	3,972 (96.5)	12,503 (96.2)
**Rural**	255 (3.9)	70 (3.1)	325 (3.7)	90 (3.1)	30 (2.5)	120 (2.9)	445 (3.4)
**Missing**	20 (0.3)	< = 5 (0.2)	25 (0.3)	20 (0.7)	< = 5 (0.3)	23 (0.6)	48 (0.4)
**Economic Dependency quintile: n (%)** ^**&**^							
**1**	2,346 (35.4)	878 (38.8)	3,224 (36.3)	1,039 (35.4)	405 (34.2)	1,444 (35.1)	4,668 (35.9)
**2**	1,338 (20.2)	505 (22.3)	1,843 (20.8)	687 (23.4)	334 (28.2)	1,021 (24.8)	2,864 (22.0)
**3**	1,044 (15.8)	342 (15.1)	1,386 (15.6)	423 (14.4)	174 (14.7)	597 (14.5)	1,983 (15.3)
**4**	875 (13.2)	249 (11.0)	1,124 (12.7)	395 (13.5)	133 (11.2)	528 (12.8)	1,652 (12.7)
**5**	876 (13.2)	250 (11.1)	1,126 (12.7)	350 (11.9)	128 (10.8)	478 (11.6)	1,604 (12.3)
**Missing**	140 (2.1)	38 (1.7)	178 (2.0)	38 (1.3)	9 (0.8)	47 (1.1)	225 (1.7)

SD: Standard deviation

*Within 3 months of HIV diagnosis

**Beyond 3 months of HIV diagnosis or no test

^&^ Higher is more deprived.

### Two-year mortality

**Fig 1:**
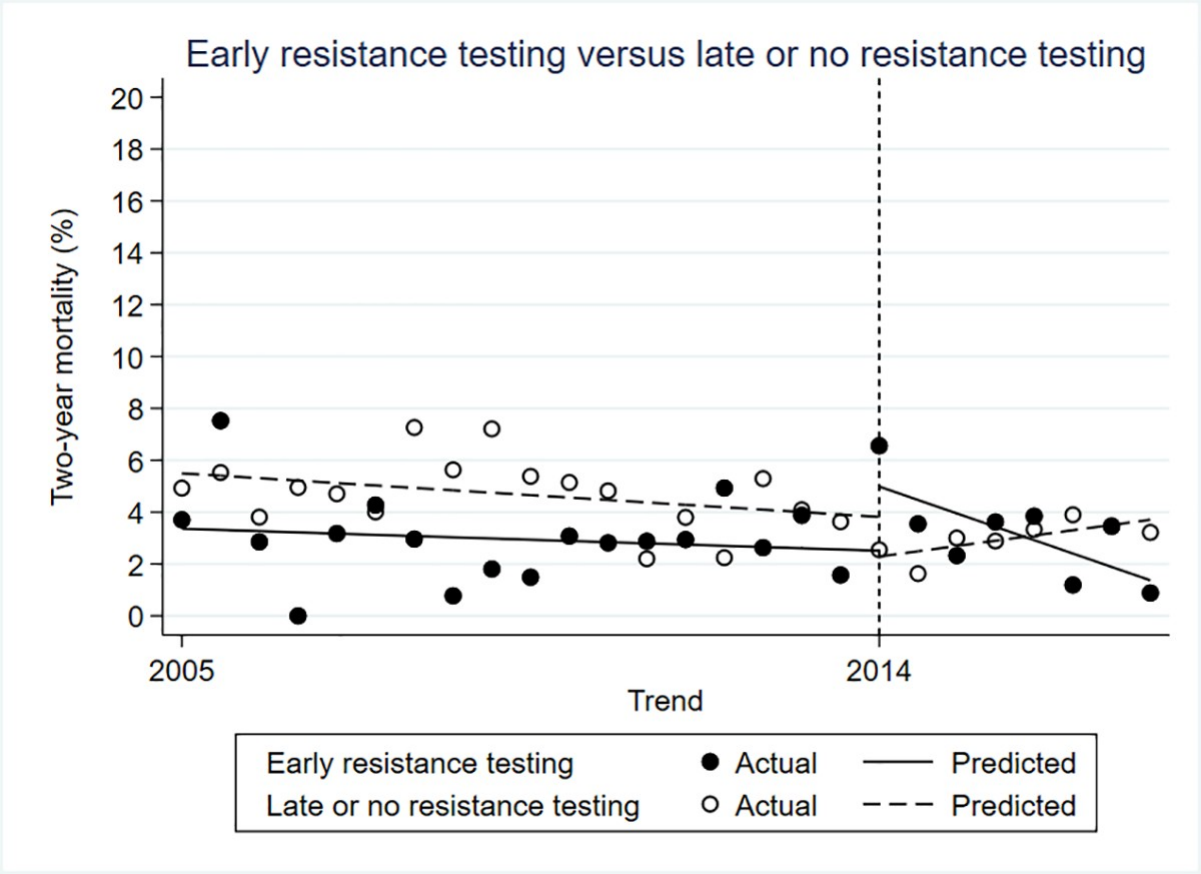
Controlled interrupted time series for two-year mortality.

There was a 2% lower mortality in the intervention group prior to the policy change (estimated β = -0.022; 95% confidence interval [CI] -0.041 to -0.003; p = 0.030). Immediately after the policy change, there was a 5% higher mortality in the intervention group (estimated β = 0.048; 95% CI 0.013 to 0.083; p = 0.010). However, in the period following the policy change, mortality dropped by 0.8% every period, compared to the control group (estimated β = -0.008; 95% CI-0.014 to -0.002; p = 0.020) (Fig 1).

### Two-year hospitalization

**Fig 2:**
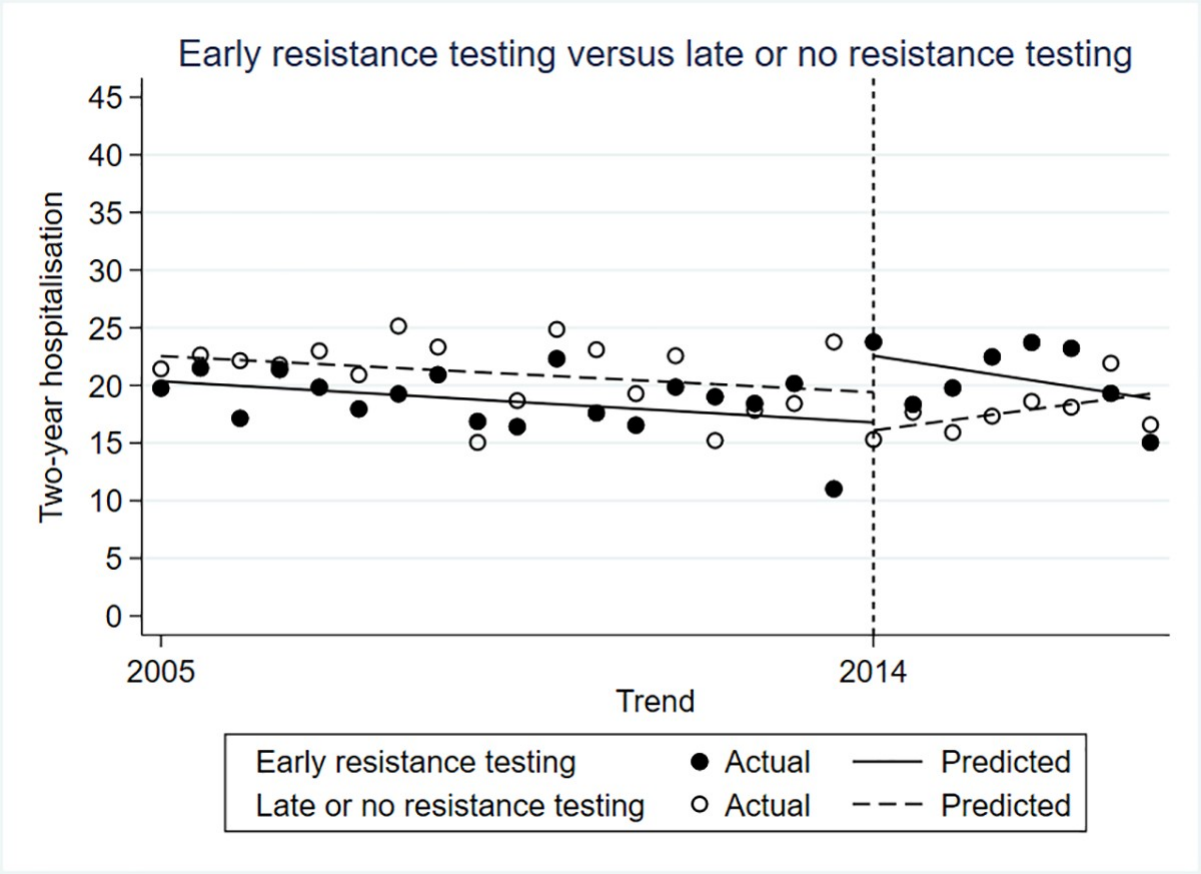
Controlled interrupted time series for two-year hospitalizations.

The difference in hospitalization rate between the control and intervention groups was not statistically significant prior to the policy change (β = -0.022; 95% CI -0.058 to -0.015; p = 0.249). Immediately after the policy change there was a 10% higher hospitalization rate in the intervention group compared to the control (β = 0.101; 95% CI 0.034 to 0.0167; p = 0.005), but no difference in the trends in hospitalization in the periods after (β = -0.010; 95% CI -0.022 to 0.002) (Fig 2).

### Two-year emergency department visits

**Fig 3:**
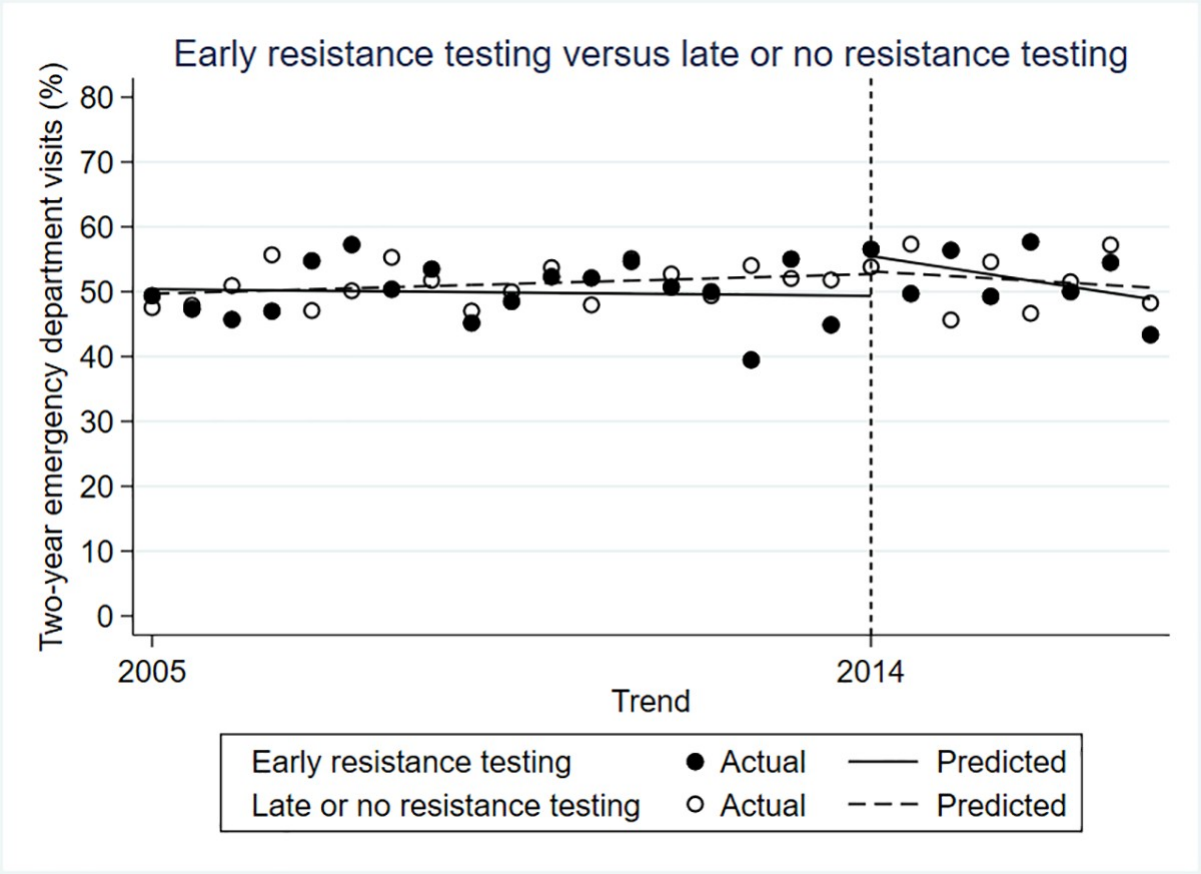
Controlled interrupted time series for two-year emergency department visits.

There was no significant difference between the intervention and control groups in the rate of emergency department visits before (β = 0.009; 95% CI-0.047 to 0.065; p = 0.751), immediately after (β = 0.061; 95% CI -0.042 to 0.164; p = 0.253) or in the period following the policy change (β = -0.004; 95% CI -0.022 to 0.015; p = 0.700) (Fig 3).

Full model details are presented in Table 2.

**Table 2:** Model parameters for interrupted time series of early resistance testing compared to late or no resistance testing.

Parameter	Parameter description	Estimated β (95% CI)	p-value
**Mortality**			
β0	control pre-intercept	0.056 (0.042 to 0.069)	<0.001
β1	control pre-slope	-0.001 (-0.002 to 0.000)	0.149
β2	control post-level change	-0.018 (-0.043 to 0.006)	0.154
β3	control post-slope change	0.003 (-0.001 to 0.007)	0.194
β4	treatment/control pre-level difference	-0.022 (-0.041 to -0.003)	0.030
β5	treatment/control pre-slope difference	0.000 (-0.001 to 0.002)	0.610
β6	treatment/control post-level difference	0.048 (0.013 to 0.083)	0.010
β7	treatment/control post-change in slope difference	-0.008 (-0.014 to -0.002)	0.020
**Hospitalizations**			
β0	control pre-intercept	0.227 (0.201 to 0.253)	<0.001
β1	control pre-slope	-0.002 (-0.004 to 0.001)	0.157
β2	control post-level change	-0.039 (-0.087 to 0.008)	0.108
β3	control post-slope change	0.006 (-0.002 to 0.015)	0.150
β4	treatment/control pre-level difference	-0.022 (-0.058 to -0.015)	0.249
β5	treatment/control pre-slope difference	0.000 (-0.004 to 0.003)	0.890
β6	treatment/control post-level difference	0.101 (0.034 to 0.167)	0.005
β7	treatment/control post-change in slope difference	-0.010 (-0.022 to 0.002)	0.120
**Emergency department visits**			
β0	control pre-intercept	0.495 (0.455 to 0.535)	<0.001
β1	control pre-slope	0.002 (-0.002 to 0.005)	0.373
β2	control post-level change	0.009 (-0.064 to 0.082)	0.804
β3	control post-slope change	-0.005 (-0.018 to 0.008)	0.435
β4	treatment/control pre-level difference	0.009 (-0.047 to 0.065)	0.751
β5	treatment/control pre-slope difference	-0.002 (-0.007 to 0.003)	0.400
β6	treatment/control post-level difference	0.061 (-0.042 to 0.164)	0.253
β7	treatment/control post-change in slope difference	-0.004 (-0.022 to 0.015)	0.700

### Sensitivity analysis

We identified 3 outlier data points and conducted a sensitivity analysis excluding these extreme data points, generating similar results. We also estimated our outcomes quarterly instead of bi-annually and got the same results. The results of the later sensitivity analyses are shown in S4 Appendix.

## Discussion

In this cohort of 12,996 people with HIV in Ontario followed up for 13 years (2005 and 2019), we found that the implementation of a drug resistance testing policy of testing prior to treatment initiation in 2014 reduced two-year mortality, but not hospitalizations and emergency department visits. These findings are in line with American and European treatment guidelines that recommend resistance testing prior to initiating treatment in people with HIV [31, 32], and provide further additional evidence to support its use.

Our findings differ from one cohort study comparing the use of resistance testing to no resistance testing in European and Canadian patients with HIV which reported improvements in virological response, but no change in progression to AIDS or death at 5 years. The authors reported that the prevalence of TDR was low (5.8%) [33]. The population-level effect of resistance testing may be smaller if the prevalence of TDR is low.

We noted a 5% increase in mortality and a 10% increase in hospitalizations immediately after the policy. This was an unexpected finding. We hypothesize that with the expanded policy, more people diagnosed in late HIV infection (who have a higher underlying risk of death) underwent resistance testing than prior to the implementation of the policy. This might also be because the resistance testing policy led to more people who live in low income neighbourhoods or rural areas (who already have a higher underlying mortality rate) [29] to get resistance tests, this could increase the rates of mortality and hospitalization immediately after the policy. Confirmation of this hypothesis would require HIV clinical histories including CD4 cell counts, which were unavailable in health administrative data for this analysis.

Further research is required to understand why in the presence of a policy recommending routine resistance testing, some people do not get a resistance test. It is possible that there are provider or patient level factors that influence the use of resistance testing to inform treatment. Also, a resistance test would not be conducted if the viral load is ≤1000 copies/mL [18]. Despite universal access to health care in Canada, there are disparities in access to resistance testing [28]. For example, Indigenous people in British Columbia [28], men who have sex with men, and people who inject drugs in Ontario are less likely to have a drug resistance test [27, 34]. On the other hand, in British Columbia, males were more likely to be tested for drug resistance before initiating therapy than females [28], but Indigenous persons were less likely to be tested and when tested, were more likely to be drug resistant than non-Indigenous persons [11]. These differences likely, in part, have to do with complex social and structural factors, including stigma that interact to shape access to healthcare, including access to, and uptake of resistance testing [35]. Sex differences in resistance testing signal the need for researchers to conduct sex and gender-based analyses to examine how sex characteristics and/or gender norms, gender roles and institutionalized gender influence the barriers to uptake of practices such as drug resistance testing [36].

This study is not without limitations. First, the role of resistance testing in improving outcomes of HIV care depends in part on the prevalence of TDR, the efficacy of ART and adherence to ART, none of which was captured in this study. However, we do not believe that these factors would have been substantially different in the ‘no or late resistance testing’ group, and changes over time are factored into pre-post trend comparisons. We would also like to clarify that these factors, though relevant to individual patients, play a limited role in population-level inferences for which interrupted time series are intended. Further, given the relative novelty of this policy (2014), we were unable to estimate long term outcomes, i.e., 2-year mortality could not be estimated for patients enrolled beyond 2017. These results must also be viewed in the context of recent changes in ART such as the availability of integrase strand inhibitors [37], which exhibit a high barrier to resistance, as well as the widespread use of pre-exposure prophylaxis which may exacerbate the incidence of drug resistance [38].

Some of the strengths of this work include the use of a control group, the use of policy relevant and patient important outcomes, and its novelty. To the best of our knowledge this is the first study to investigate the population-level effect of a pre-treatment resistance testing policy and show the benefits of early routine resistance testing for everyone diagnosed with HIV. These findings would be of interest to countries that already have such policies in place and for countries that need to make evidence-informed decisions about implementing routine resistance testing.

Future work should incorporate relevant patient level characteristics that influence engagement in care, access to care and risk of TDR. Research should also examine physician-level factors and barriers associated with uptake of resistance testing, and if there is a need for specialized approaches to engage women and Indigenous persons.

Resistance testing prior to initiating therapy may lead to fewer deaths, but no difference was shown in emergency room visits or hospitalizations within 2 years. Exploring the individual-level impacts of drug resistance testing and the characteristics of patients who benefit the most from resistance testing may inform tailored approaches to care and provide further impetus for strengthening the HIV care cascade.

## Supporting information

S1 Appendix Sample size and power. (DOCX)

S2 Appendix Full controlled interrupted time series model. (DOCX)

S3 Appendix Approaches used for autocorrelation. (DOCX)

S4 Appendix Sensitivity analyses quarterly. (DOCX)

S1 File STROBE statement—checklist of items that should be included in reports of observational studies. (DOC)
