# Supplementary material for: The impact of routine HIV drug resistance testing in Ontario: A controlled interrupted time series study
Source: PLoS One. 2021 Apr 2;16(4):e0246766. doi: 10.1371/journal.pone.0246766 (PMC8018617; doi:10.1371/journal.pone.0246766)
Supplement: S1 Appendix — (DOCX) [file pone.0246766.s001.docx]

**S1 Appendix: Sample size and power:**

Sampling requirements were computed for mortality. With 18 pre-intervention time-points (bi-annually), assuming 95% confidence intervals and a level of significance of $\alpha=$0.05, this study has >90% power to detect a 4% absolute reduction in mortality rates from 13,000 participants. These computations were done using the *itspower* command in STATA version 16.0. Estimates were drawn from pooled data from 9 trials including treatment-experienced patients.(1) The benefits (reductions in mortality) of resistance testing in a treatment-naïve population are likely to be higher and would lead to smaller sample size requirements. The available data allowed for pre- and post- intervention comparisons and between group comparisons.

**Reference:**

1. Aves T, Tambe J, Siemieniuk RA, Mbuagbaw L. Antiretroviral resistance testing in HIV-positive people. Cochrane Database Syst Rev. 2018;11:CD006495.
