# Supplementary material for: The impact of routine HIV drug resistance testing in Ontario: A controlled interrupted time series study
Source: PLoS One. 2021 Apr 2;16(4):e0246766. doi: 10.1371/journal.pone.0246766 (PMC8018617; doi:10.1371/journal.pone.0246766)
Supplement: S2 Appendix — (DOCX) [file pone.0246766.s002.docx]

**S2 Appendix: Full Controlled Interrupted time series model**

Two parameters define each segment of a time series: level and trend. The level is the value of the series at the beginning of a given time interval. The trend is the rate of change of a measure (i.e. the slope) during a segment. The intervention effect is estimated by comparing the trend of the outcome in the post-intervention period to the existing trend in the outcome in the pre-intervention period. In this segmented regression analysis, the time period is divided into pre- and post-intervention segments, and separate intercepts and slopes are estimated in each segment. Statistical tests of changes in intercepts and slopes from pre to post-intervention and between intervention and control groups are carried out. An additional adjustment to account for serial autocorrelation is performed. This arises because observations taken over time are usually correlated. The OLS models used here adjust for autocorrelation.(1)

Equation:

y = α + β1T + β2X + β3XT + β4 Z + β5 ZT + β6 ZX + β7 ZXT + ε

where Z is a dummy variable denoting treatment or control,

- T denotes the time since the start of the study
- X is a dummy variable representing the intervention
- ZT, ZX and ZXT are interaction terms for the variables above
- β0 estimates the baseline level of the outcome in the control group (control pre-intercept)
- β1 estimates the change in outcome before the intervention in the control group (control pre-slope)
- β2 estimates the change in the outcome occurring immediately after the intervention in the control group (control post-level change)
- β3 estimates the change in the outcome each time period after the policy in the control group (control post-slope change)
- β4 estimates the difference in baseline level of outcome between the treatment and control before the intervention (treatment/control pre-level difference)
- β5 estimates the difference in trends between treatment and control before the intervention (treatment/control pre-slope difference)
- β6 estimates the difference in change in outcome between the treatment and control group immediately after the intervention (treatment/control post-level difference)
- β7 difference between treatment and control groups in the trend of the outcome variable after initiation of the intervention compared with before the intervention (treatment/control post-change in slope difference)
- ε is the error term.

**Reference:**

1. Linden A. Conducting interrupted time-series analysis for single-and multiple-group comparisons. The Stata Journal. 2015;15(2):480-500.
