# Supplementary material for: The impact of routine HIV drug resistance testing in Ontario: A controlled interrupted time series study
Source: PLoS One. 2021 Apr 2;16(4):e0246766. doi: 10.1371/journal.pone.0246766 (PMC8018617; doi:10.1371/journal.pone.0246766)
Supplement: S3 Appendix — (DOCX) [file pone.0246766.s003.docx]

**S3 Appendix: Approaches used for autocorrelation**

Ordinary least squares regression analysis assumes that error terms associated with each observation (i.e. the differences between the actual outcome values and those predicted from the regression model) are uncorrelated. Since time is a predictor in segmented regression analysis, error terms of consecutive observations are often correlated, resulting in serial autocorrelation of the error terms. Correlation between adjacent datapoints is termed first-order autocorrelation; correlation between the current point and 2 time points before or after would be second-order autocorrelation and so forth. There may also be seasonal patterns in monthly time series. This is an example of higher-order autocorrelation.

Failing to correct for autocorrelation may lead to under-estimated standard errors and overestimated significance of the effects of an intervention. Autoregressive integrated moving average (ARIMA) modelling is used to correct for this. Alternatively, we use PROC AUTOREG in SAS to detect autocorrelation in time series data and control for it. PROC AUTOREG estimates an autoregressive model, and automatically tests for autocorrelations in the data, estimate autoregressive parameters to be included in the model, and estimate the final parameters with the autoregressive parameters assumed given. The following SAS code is implemented:

PROC AUTOREG data=data outset=outest;

Model outcome = T X TX / method=ml nlag=2 backstep DWPROB loglikl;

Output out=ITS p=pvar r=rvar;

Run;

This code specifies that 2 lags should be tested and parameters included in the final model if statistically significant. Line 2 specifies that these lags should be entered into the model using backward elimination to sequentially remove autoregressive parameters not significant at the 0.05 level, in order to fit the most parsimonious model. The DWPROB option specifies that a Durbin-Watson test is to be used to test for the presence of autocorrelation. The LOGLIKL option specifies that the log likelihood for the overall model be produced in order to assess the overall quality of the model. The OUT = statement produces a SAS dataset to store the predicted values and residuals from the model. The p=option specifies the full model predicted values where p is the variable for the predicted mean; and r= option is the variable for the residuals.

The output from the AUTOREG procedure includes a section for Ordinary Least Squares Estimates, Autoregressive Error Analysis, and Final Model Estimation. The final model estimation section includes the Fit Summary, Durbin-Watson Statistics, Parameter Estimates, and Parameter Estimates with AR Parameters Assumed given. PROC AUTOREG calculates diagnostics including the Durbin-Watson statistics based on an autoregressive process of order k if one specified dw=k dwprob.

To detect autocorrelation, one can visually inspect a plot of residuals against time (outputted by PROC AUTOREG). Randomly scattered residuals, without a pattern, indicate that there is no autocorrelation. Positive autocorrelation exists when consecutive residuals tend to lie on the same side of the regression line; negative autocorrelation exists when consecutive residuals tend to lie on different sides of the regression line.

The Durbin-Watson statistic tests for serial autocorrelation of the error terms in the regression model. Values close to 2.00 indicate no serious autocorrelation. Adjustment for autocorrelation involves estimating the autocorrelation parameter and including it in the segmented regression model, if necessary.

Fit of the final model is assessed by examining the residuals around the predicted regression lines. Residuals that are normally distributed and that follow no observable pattern over time indicate that the assumptions underlying the linear model are met.
