# Supplementary material for: The impact of routine HIV drug resistance testing in Ontario: A controlled interrupted time series study
Source: PLoS One. 2021 Apr 2;16(4):e0246766. doi: 10.1371/journal.pone.0246766 (PMC8018617; doi:10.1371/journal.pone.0246766)
Supplement: S4 Appendix — (DOCX) [file pone.0246766.s004.docx]

**S4 Appendix: Model parameters for interrupted time series of early resistance testing compared to late or no resistance testing with outcomes estimated quarterly**

| **Parameter** | **Parameter description** | **Estimated** β **(95% CI)** | **p-value** |
| --- | --- | --- | --- |
| **Mortality** | | | |
| β0 | control pre-intercept | 0.056 (0.044 to 0.068) | <0.001 |
| β1 | control pre-slope | 0.000 (0.000 to 0.000) | 0.126 |
| β2 | control post-level change | -0.017 (-0.041 to 0.007) | 0.138 |
| β3 | control post-slope change | 0.001 (-0.001 to 0.003) | 0.182 |
| β4 | treatment/control pre-level difference | -0.024 (-0.042 to -0.006) | 0.009 |
| β5 | treatment/control pre-slope difference | 0 (0 to 0) | 0.456 |
| β6 | treatment/control post-level difference | 0.043 (0.012 to 0.074) | 0.009 |
| β7 | treatment/control post-change in slope difference | -0.004 (-0.006 to -0.002) | 0.016 |
| **Hospitalizations** | | | |
| β0 | control pre-intercept | 0.227 (0.2 to 0.254) | <0.001 |
| β1 | control pre-slope | -0.001 (-0.003 to 0.001) | 0.183 |
| β2 | control post-level change | -0.037 (-0.086 to 0.012) | 0.144 |
| β3 | control post-slope change | 0.003 (-0.001 to 0.007) | 0.188 |
| β4 | treatment/control pre-level difference | -0.025 (-0.062 to 0.012) | 0.199 |
| β5 | treatment/control pre-slope difference | 0 (-0.002 to 0.002) | 0.941 |
| β6 | treatment/control post-level difference | 0.095 (0.026 to 0.164) | 0.008 |
| β7 | treatment/control post-change in slope difference | -0.005 (-0.011 to 0.001) | 0.143 |
| **Emergency department visits** | | | |
| β0 | control pre-intercept | 0.494 (0.457 to 0.531) | <0.001 |
| β1 | control pre-slope | 0.001 (-0.001 to 0.003) | 0.323 |
| β2 | control post-level change | 0.007 (-0.062 to 0.076) | 0.833 |
| β3 | control post-slope change | -0.003 (-0.009 to 0.003) | 0.387 |
| β4 | treatment/control pre-level difference | 0.007 (-0.048 to 0.062) | 0.787 |
| β5 | treatment/control pre-slope difference | -0.001 (-0.003 to 0.001) | 0.412 |
| β6 | treatment/control post-level difference | 0.053 (-0.045 to 0.151) | 0.296 |
| β7 | treatment/control post-change in slope difference | -0.001 (-0.011 to 0.009) | 0.911 |
